# Supplementary material for: Pyruvate Dehydrogenase Kinase 1 inhibition mediated oxidative phosphorylation enhancement in cartilage promotes osteoarthritis progression
Source: BMC Musculoskelet Disord. 2023 Jul 20;24:597. doi: 10.1186/s12891-023-06585-6 (PMC10357736; doi:10.1186/s12891-023-06585-6)
Supplement: Supplementary file 1 — Additional file 1. [file 12891_2023_6585_MOESM1_ESM.zip › BP.Gsea.1653623667859/GOBP_EPITHELIAL_CELL_APOPTOTIC_PROCESS.html]

Details for gene set GOBP\_EPITHELIAL\_CELL\_APOPTOTIC\_PROCESS[GSEA]

|  || Dataset | 55457\_collapsed\_to\_symbols.cls.cls #OA\_versus\_normal.cls.cls #OA\_versus\_normal\_repos |
| Phenotype | cls.cls#OA\_versus\_normal\_repos |
| Upregulated in class | normal |
| GeneSet | GOBP\_EPITHELIAL\_CELL\_APOPTOTIC\_PROCESS |
| Enrichment Score (ES) | -0.4056539 |
| Normalized Enrichment Score (NES) | -1.7772859 |
| Nominal p-value | 0.0 |
| FDR q-value | 0.11424009 |
| FWER p-Value | 0.993 |
Table: GSEA Results Summary

  

Fig 1: Enrichment plot: GOBP\_EPITHELIAL\_CELL\_APOPTOTIC\_PROCESS      
 Profile of the Running ES Score & Positions of GeneSet Members on the Rank Ordered List

  

| SYMBOL | TITLE | RANK IN GENE LIST | RANK METRIC SCORE | RUNNING ES | CORE ENRICHMENT || 1 | DNMT3A | DNA methyltransferase 3 alpha [Source:HGNC Symbol;Acc:HGNC:2978] | 406 | 0.704 | -0.0093 | No |
| 2 | RYR2 | ryanodine receptor 2 [Source:HGNC Symbol;Acc:HGNC:10484] | 828 | 0.561 | -0.0244 | No |
| 3 | NEUROD1 | neuronal differentiation 1 [Source:HGNC Symbol;Acc:HGNC:7762] | 1008 | 0.515 | -0.0220 | No |
| 4 | ANGPT1 | angiopoietin 1 [Source:HGNC Symbol;Acc:HGNC:484] | 1467 | 0.433 | -0.0440 | No |
| 5 | GPER1 | G protein-coupled estrogen receptor 1 [Source:HGNC Symbol;Acc:HGNC:4485] | 1677 | 0.402 | -0.0475 | No |
| 6 | ITGA4 | integrin subunit alpha 4 [Source:HGNC Symbol;Acc:HGNC:6140] | 1854 | 0.379 | -0.0492 | No |
| 7 | GAS6 | growth arrest specific 6 [Source:HGNC Symbol;Acc:HGNC:4168] | 1942 | 0.368 | -0.0443 | No |
| 8 | AKR1C3 | aldo-keto reductase family 1 member C3 [Source:HGNC Symbol;Acc:HGNC:386] | 1996 | 0.361 | -0.0370 | No |
| 9 | NFE2L2 | "nuclear factor, erythroid 2 like 2 [Source:HGNC Symbol;Acc:HGNC:7782]" | 2034 | 0.357 | -0.0285 | No |
| 10 | TNF | tumor necrosis factor [Source:HGNC Symbol;Acc:HGNC:11892] | 2098 | 0.350 | -0.0223 | No |
| 11 | SCG2 | secretogranin II [Source:HGNC Symbol;Acc:HGNC:10575] | 2156 | 0.343 | -0.0158 | No |
| 12 | EXOC5 | exocyst complex component 5 [Source:HGNC Symbol;Acc:HGNC:10696] | 2243 | 0.334 | -0.0119 | No |
| 13 | CASP6 | caspase 6 [Source:HGNC Symbol;Acc:HGNC:1507] | 2427 | 0.311 | -0.0163 | No |
| 14 | NDNF | neuron derived neurotrophic factor [Source:HGNC Symbol;Acc:HGNC:26256] | 2988 | 0.251 | -0.0521 | No |
| 15 | CD160 | CD160 molecule [Source:HGNC Symbol;Acc:HGNC:17013] | 3158 | 0.234 | -0.0578 | No |
| 16 | SEMA5A | semaphorin 5A [Source:HGNC Symbol;Acc:HGNC:10736] | 3308 | 0.220 | -0.0625 | No |
| 17 | FGB | fibrinogen beta chain [Source:HGNC Symbol;Acc:HGNC:3662] | 3721 | 0.183 | -0.0888 | No |
| 18 | BAD | BCL2 associated agonist of cell death [Source:HGNC Symbol;Acc:HGNC:936] | 3788 | 0.178 | -0.0883 | No |
| 19 | FASLG | Fas ligand [Source:HGNC Symbol;Acc:HGNC:11936] | 3931 | 0.162 | -0.0943 | No |
| 20 | JAK2 | Janus kinase 2 [Source:HGNC Symbol;Acc:HGNC:6192] | 4183 | 0.140 | -0.1094 | No |
| 21 | IL11 | interleukin 11 [Source:HGNC Symbol;Acc:HGNC:5966] | 4327 | 0.126 | -0.1166 | No |
| 22 | MAPK7 | mitogen-activated protein kinase 7 [Source:HGNC Symbol;Acc:HGNC:6880] | 4396 | 0.121 | -0.1180 | No |
| 23 | EDNRA | endothelin receptor type A [Source:HGNC Symbol;Acc:HGNC:3179] | 4663 | 0.099 | -0.1357 | No |
| 24 | COL4A3 | collagen type IV alpha 3 chain [Source:HGNC Symbol;Acc:HGNC:2204] | 4774 | 0.089 | -0.1414 | No |
| 25 | TCF7L2 | transcription factor 7 like 2 [Source:HGNC Symbol;Acc:HGNC:11641] | 5034 | 0.070 | -0.1594 | No |
| 26 | YAP1 | Yes1 associated transcriptional regulator [Source:HGNC Symbol;Acc:HGNC:16262] | 5041 | 0.070 | -0.1577 | No |
| 27 | E2F1 | E2F transcription factor 1 [Source:HGNC Symbol;Acc:HGNC:3113] | 5137 | 0.061 | -0.1632 | No |
| 28 | SFRP4 | secreted frizzled related protein 4 [Source:HGNC Symbol;Acc:HGNC:10778] | 5443 | 0.037 | -0.1858 | No |
| 29 | CD40LG | CD40 ligand [Source:HGNC Symbol;Acc:HGNC:11935] | 5529 | 0.030 | -0.1915 | No |
| 30 | NUPR1 | "nuclear protein 1, transcriptional regulator [Source:HGNC Symbol;Acc:HGNC:29990]" | 5616 | 0.023 | -0.1975 | No |
| 31 | BID | BH3 interacting domain death agonist [Source:HGNC Symbol;Acc:HGNC:1050] | 5715 | 0.014 | -0.2047 | No |
| 32 | TERT | telomerase reverse transcriptase [Source:HGNC Symbol;Acc:HGNC:11730] | 5773 | 0.010 | -0.2088 | No |
| 33 | MDK | midkine [Source:HGNC Symbol;Acc:HGNC:6972] | 5828 | 0.007 | -0.2128 | No |
| 34 | CAST | calpastatin [Source:HGNC Symbol;Acc:HGNC:1515] | 5941 | -0.001 | -0.2215 | No |
| 35 | PDCD4 | programmed cell death 4 [Source:HGNC Symbol;Acc:HGNC:8763] | 5961 | -0.002 | -0.2230 | No |
| 36 | FGG | fibrinogen gamma chain [Source:HGNC Symbol;Acc:HGNC:3694] | 5997 | -0.004 | -0.2256 | No |
| 37 | RB1 | RB transcriptional corepressor 1 [Source:HGNC Symbol;Acc:HGNC:9884] | 6013 | -0.006 | -0.2266 | No |
| 38 | SIX3 | SIX homeobox 3 [Source:HGNC Symbol;Acc:HGNC:10889] | 6356 | -0.032 | -0.2523 | No |
| 39 | IL4 | interleukin 4 [Source:HGNC Symbol;Acc:HGNC:6014] | 6673 | -0.054 | -0.2752 | No |
| 40 | BCL2L1 | BCL2 like 1 [Source:HGNC Symbol;Acc:HGNC:992] | 6698 | -0.055 | -0.2754 | No |
| 41 | CD40 | CD40 molecule [Source:HGNC Symbol;Acc:HGNC:11919] | 6825 | -0.065 | -0.2831 | No |
| 42 | KRT18 | keratin 18 [Source:HGNC Symbol;Acc:HGNC:6430] | 7027 | -0.082 | -0.2962 | No |
| 43 | RAMP2 | receptor activity modifying protein 2 [Source:HGNC Symbol;Acc:HGNC:9844] | 7247 | -0.099 | -0.3102 | No |
| 44 | ARRB2 | arrestin beta 2 [Source:HGNC Symbol;Acc:HGNC:712] | 7255 | -0.100 | -0.3076 | No |
| 45 | PLA2R1 | phospholipase A2 receptor 1 [Source:HGNC Symbol;Acc:HGNC:9042] | 7321 | -0.103 | -0.3094 | No |
| 46 | TNIP2 | TNFAIP3 interacting protein 2 [Source:HGNC Symbol;Acc:HGNC:19118] | 7659 | -0.131 | -0.3315 | No |
| 47 | SERPINB13 | serpin family B member 13 [Source:HGNC Symbol;Acc:HGNC:8944] | 7917 | -0.149 | -0.3469 | No |
| 48 | PIK3CG | "phosphatidylinositol-4,5-bisphosphate 3-kinase catalytic subunit gamma [Source:HGNC Symbol;Acc:HGNC:8978]" | 7953 | -0.152 | -0.3448 | No |
| 49 | E2F2 | E2F transcription factor 2 [Source:HGNC Symbol;Acc:HGNC:3114] | 8090 | -0.162 | -0.3502 | No |
| 50 | PLA2G1B | phospholipase A2 group IB [Source:HGNC Symbol;Acc:HGNC:9030] | 8232 | -0.173 | -0.3557 | No |
| 51 | TGFBR2 | transforming growth factor beta receptor 2 [Source:HGNC Symbol;Acc:HGNC:11773] | 8241 | -0.174 | -0.3508 | No |
| 52 | WFS1 | wolframin ER transmembrane glycoprotein [Source:HGNC Symbol;Acc:HGNC:12762] | 8299 | -0.178 | -0.3496 | No |
| 53 | GSN | gelsolin [Source:HGNC Symbol;Acc:HGNC:4620] | 8507 | -0.194 | -0.3596 | No |
| 54 | ATF2 | activating transcription factor 2 [Source:HGNC Symbol;Acc:HGNC:784] | 8592 | -0.201 | -0.3598 | No |
| 55 | TEK | TEK receptor tyrosine kinase [Source:HGNC Symbol;Acc:HGNC:11724] | 8623 | -0.203 | -0.3557 | No |
| 56 | CD248 | CD248 molecule [Source:HGNC Symbol;Acc:HGNC:18219] | 8816 | -0.219 | -0.3637 | No |
| 57 | IL10 | interleukin 10 [Source:HGNC Symbol;Acc:HGNC:5962] | 8855 | -0.221 | -0.3597 | No |
| 58 | FGA | fibrinogen alpha chain [Source:HGNC Symbol;Acc:HGNC:3661] | 8880 | -0.223 | -0.3544 | No |
| 59 | ABL1 | "ABL proto-oncogene 1, non-receptor tyrosine kinase [Source:HGNC Symbol;Acc:HGNC:76]" | 9081 | -0.238 | -0.3625 | No |
| 60 | EIF2S1 | eukaryotic translation initiation factor 2 subunit alpha [Source:HGNC Symbol;Acc:HGNC:3265] | 9310 | -0.256 | -0.3721 | No |
| 61 | ICAM1 | intercellular adhesion molecule 1 [Source:HGNC Symbol;Acc:HGNC:5344] | 9740 | -0.296 | -0.3962 | Yes |
| 62 | CCL2 | C-C motif chemokine ligand 2 [Source:HGNC Symbol;Acc:HGNC:10618] | 9819 | -0.303 | -0.3927 | Yes |
| 63 | CAPN10 | calpain 10 [Source:HGNC Symbol;Acc:HGNC:1477] | 9859 | -0.307 | -0.3860 | Yes |
| 64 | CDH5 | cadherin 5 [Source:HGNC Symbol;Acc:HGNC:1764] | 9946 | -0.316 | -0.3827 | Yes |
| 65 | PAK4 | p21 (RAC1) activated kinase 4 [Source:HGNC Symbol;Acc:HGNC:16059] | 10004 | -0.322 | -0.3769 | Yes |
| 66 | SERPINE1 | serpin family E member 1 [Source:HGNC Symbol;Acc:HGNC:8583] | 10284 | -0.349 | -0.3876 | Yes |
| 67 | FGF21 | fibroblast growth factor 21 [Source:HGNC Symbol;Acc:HGNC:3678] | 10341 | -0.355 | -0.3807 | Yes |
| 68 | HIPK1 | homeodomain interacting protein kinase 1 [Source:HGNC Symbol;Acc:HGNC:19006] | 10391 | -0.360 | -0.3731 | Yes |
| 69 | PDX1 | pancreatic and duodenal homeobox 1 [Source:HGNC Symbol;Acc:HGNC:6107] | 10409 | -0.362 | -0.3629 | Yes |
| 70 | KRIT1 | KRIT1 ankyrin repeat containing [Source:HGNC Symbol;Acc:HGNC:1573] | 10575 | -0.381 | -0.3636 | Yes |
| 71 | STK3 | serine/threonine kinase 3 [Source:HGNC Symbol;Acc:HGNC:11406] | 10656 | -0.388 | -0.3575 | Yes |
| 72 | SRSF6 | serine and arginine rich splicing factor 6 [Source:HGNC Symbol;Acc:HGNC:10788] | 10659 | -0.389 | -0.3453 | Yes |
| 73 | PLCG1 | phospholipase C gamma 1 [Source:HGNC Symbol;Acc:HGNC:9065] | 10722 | -0.395 | -0.3376 | Yes |
| 74 | HLA-G | "major histocompatibility complex, class I, G [Source:HGNC Symbol;Acc:HGNC:4964]" | 10869 | -0.413 | -0.3359 | Yes |
| 75 | MAP3K5 | mitogen-activated protein kinase kinase kinase 5 [Source:HGNC Symbol;Acc:HGNC:6857] | 10923 | -0.421 | -0.3266 | Yes |
| 76 | JAG2 | jagged canonical Notch ligand 2 [Source:HGNC Symbol;Acc:HGNC:6189] | 11039 | -0.435 | -0.3218 | Yes |
| 77 | GATA2 | GATA binding protein 2 [Source:HGNC Symbol;Acc:HGNC:4171] | 11058 | -0.439 | -0.3092 | Yes |
| 78 | STK4 | serine/threonine kinase 4 [Source:HGNC Symbol;Acc:HGNC:11408] | 11074 | -0.441 | -0.2964 | Yes |
| 79 | ARF6 | ADP ribosylation factor 6 [Source:HGNC Symbol;Acc:HGNC:659] | 11084 | -0.442 | -0.2830 | Yes |
| 80 | PPARA | peroxisome proliferator activated receptor alpha [Source:HGNC Symbol;Acc:HGNC:9232] | 11110 | -0.445 | -0.2708 | Yes |
| 81 | BMPR2 | bone morphogenetic protein receptor type 2 [Source:HGNC Symbol;Acc:HGNC:1078] | 11174 | -0.454 | -0.2613 | Yes |
| 82 | FOXO3 | forkhead box O3 [Source:HGNC Symbol;Acc:HGNC:3821] | 11305 | -0.472 | -0.2565 | Yes |
| 83 | ANGPTL4 | angiopoietin like 4 [Source:HGNC Symbol;Acc:HGNC:16039] | 11389 | -0.484 | -0.2476 | Yes |
| 84 | KDR | kinase insert domain receptor [Source:HGNC Symbol;Acc:HGNC:6307] | 11454 | -0.496 | -0.2368 | Yes |
| 85 | THBS1 | thrombospondin 1 [Source:HGNC Symbol;Acc:HGNC:11785] | 11498 | -0.501 | -0.2242 | Yes |
| 86 | PRKAA1 | protein kinase AMP-activated catalytic subunit alpha 1 [Source:HGNC Symbol;Acc:HGNC:9376] | 11499 | -0.501 | -0.2083 | Yes |
| 87 | PDPK1 | 3-phosphoinositide dependent protein kinase 1 [Source:HGNC Symbol;Acc:HGNC:8816] | 11839 | -0.557 | -0.2170 | Yes |
| 88 | PIAS4 | protein inhibitor of activated STAT 4 [Source:HGNC Symbol;Acc:HGNC:17002] | 12060 | -0.610 | -0.2148 | Yes |
| 89 | CFLAR | CASP8 and FADD like apoptosis regulator [Source:HGNC Symbol;Acc:HGNC:1876] | 12079 | -0.614 | -0.1967 | Yes |
| 90 | IL13 | interleukin 13 [Source:HGNC Symbol;Acc:HGNC:5973] | 12173 | -0.636 | -0.1837 | Yes |
| 91 | GATA3 | GATA binding protein 3 [Source:HGNC Symbol;Acc:HGNC:4172] | 12276 | -0.670 | -0.1704 | Yes |
| 92 | HMOX1 | heme oxygenase 1 [Source:HGNC Symbol;Acc:HGNC:5013] | 12421 | -0.714 | -0.1589 | Yes |
| 93 | PRKCI | protein kinase C iota [Source:HGNC Symbol;Acc:HGNC:9404] | 12444 | -0.723 | -0.1377 | Yes |
| 94 | IL6 | interleukin 6 [Source:HGNC Symbol;Acc:HGNC:6018] | 12460 | -0.730 | -0.1156 | Yes |
| 95 | RGCC | regulator of cell cycle [Source:HGNC Symbol;Acc:HGNC:20369] | 12478 | -0.740 | -0.0934 | Yes |
| 96 | TNFAIP3 | TNF alpha induced protein 3 [Source:HGNC Symbol;Acc:HGNC:11896] | 12631 | -0.814 | -0.0794 | Yes |
| 97 | IGF1R | insulin like growth factor 1 receptor [Source:HGNC Symbol;Acc:HGNC:5465] | 12689 | -0.855 | -0.0567 | Yes |
| 98 | ZFP36 | ZFP36 ring finger protein [Source:HGNC Symbol;Acc:HGNC:12862] | 12846 | -1.052 | -0.0354 | Yes |
| 99 | ZFP36L1 | ZFP36 ring finger protein like 1 [Source:HGNC Symbol;Acc:HGNC:1107] | 12888 | -1.244 | 0.0009 | Yes |
Table: GSEA details [plain text format]

  

Fig 2: GOBP\_EPITHELIAL\_CELL\_APOPTOTIC\_PROCESS      
 Blue-Pink O' Gram in the Space of the Analyzed GeneSet

  

Fig 3: GOBP\_EPITHELIAL\_CELL\_APOPTOTIC\_PROCESS: Random ES distribution      
 Gene set null distribution of ES for **GOBP\_EPITHELIAL\_CELL\_APOPTOTIC\_PROCESS**
